# Update on NKCC2 regulation in the thick ascending limb (TAL) by membrane trafficking, phosphorylation, and protein-protein interactions

**DOI:** 10.3389/fphys.2024.1508806

**Published:** 2024-12-09

**Authors:** Dipak Maskey, Jessica Granados Pineda, Pablo A. Ortiz

**Affiliations:** ^1^ Department of Internal Medicine, Hypertension and Vascular Research Division, Henry ford hospital, Detroit, MI, United States; ^2^ Department of Physiology, Integrative Bioscience Center, Wayne State University, Detroit, MI, United States

**Keywords:** thick ascending limb, SLC12A1, NKCC2, protein-protein interactions, proteomics

## Abstract

**Purpose of review:**

The thick ascending limb (TAL) of loop of Henle is essential for NaCl, calcium and magnesium homeostasis, pH balance and for urine concentration. NKCC2 is the main transporter for NaCl reabsorption in the TAL and its regulation is very complex. There have been recent advancements toward understanding how NKCC2 is regulated by protein trafficking, protein-protein interaction, and phosphorylation/dephosphorylation. Here, we update the latest molecular mechanisms and players that control NKCC2 function, which gives an increasingly complex picture of NKKC2 regulation in the apical membrane of the TAL.

**Recent Findings:**

Protein-protein interactions are required as a regulatory mechanism in many cellular processes. A handful of proteins have been recently identified as an interacting partner of NKCC2, which play major roles in regulating NKCC2 trafficking and activity. New players in NKCC2 internalization and trafficking have been identified. NKCC2 activity is also regulated by kinases and phosphatases, and there have been developments in that area as well.

**Summary:**

Here we review the current understanding of apical trafficking of NKCC2 in the thick ascending limb (TAL) which is tightly controlled by protein-protein interactions, protein turnover and by phosphorylation and dephosphorylation. We discuss new proteins and processes that regulate NKCC2 that have physiological and pathological significance.

## Highlights

✓ NKCC2 at the apical membrane is a key regulator of salt reabsorption and water homeostasis.
✓ Protein-protein interactions mediate trafficking that controls NKCC2 surface expression and its functional activity.
✓ Post-translational modification of NKCC2 alters its trafficking steps.

## Introduction

In the kidney, the thick ascending limb (TAL) plays an essential role to control NaCl homeostasis, calcium and magnesium reabsorption, extracellular fluid volume, and pH balance (Zacchia et al., 2018). In the TAL, the major luminal Na^+^ transport pathway is provided by the Na^+^-K^+^-2Cl^-^ cotransporter type 2 (NKCC2), which belongs to the member of the superfamily of electroneutral cation-coupled cotransporters (CCCs) encoded by the solute carriers family 12A (SLC12A1) genes (Hebert and Andreoli, 1984; Molony et al., 1989; Gamba et al., 1994). Intracellular N- and C-terminal domains in CCCs play a role transport and trafficking activities while transmembrane domains of CCCs are responsible for ion translocation (Markadieu and Delpire, 2014). NKCC2 is exclusively expressed in the TAL and macula densa cells. In the TAL, this cotransporter is the main gateway for NaCl reabsorption *via* the apical membrane (Nielsen et al., 1998; Bazúa-Valenti et al., 2016) thereby playing an essential role in the TAL and renal function. In the macula densa, it acts as a sensor of luminal NaCl concentration and initiates the signaling required for tubulo-glomerular feedback (TGF) when NaCl concentration is low. While NKCC2 itself does not directly control plasma osmolality nor final urine osmolality, NKCC2-mediated NaCl reabsorption by the TAL is essential for water reabsorption because it maintains a high interstitial osmolarity. The TAL is one of the most water-impermeant segments in the nephron and its water permeability is not increased by AVP. As such, NaCl reabsorption through NKCC2 dilutes the forming urine in the tubule lumen while increasing interstitial osmolality. A high interstitial osmolality (ranging from 350 to 1,000 mOsm) is necessary for countercurrent multiplication and water reabsorption in the thin descending limb and water reabsorption by the collecting ducts (in the presence of vasopressin) (Layton, 2011; Layton and Layton, 2011; Gamba and Friedman, 2009; Ares et al., 2011). Therefore, NKCC2 activity is crucial for NaCl conservation, interstitial osmolality, regulating water balance, and blood pressure (Ares et al., 2011).

NKCC2-dependent NaCl entry from the tubule lumen is driven by the electrochemical gradient generated by basolateral Na-K-ATPase. Intracellular Cl exits the cell via basolateral chloride channels (CLCKa, CLCKb), K/Cl cotransporters (KCC4) while apical K recycling occurs *via* ROMK channels across the apical membrane. All these components are required to complete net transcellular transport of NaCl in the TAL (Castrop and Schnermann, 2008). Loss-of-function mutations in NKCC2 have illustrated the fundamental role of NKCC2 in human physiology and blood pressure regulation. These inactivating mutations cause Bartter syndrome type I, characterized by hypokalemic alkalosis, hypomagnesemia, hypercalciuria, excessive loss of urine NaCl, inability to concentrate urine, polyuria, and critically low blood pressure (Gamba and Friedman, 2009; Simon et al., 1996; Haas and Forbush, 1998; Takahashi et al., 2000; Welling, 2014; Gamba, 2005). At least six homozygous mutations (G193R, A267S, G319R, A508T, del526N and Y998X) have been reported in NKCC2 in patients with Barter Syndrome type I. Similarly, genetic deletion of NKCC2 in mice leads to death unless salt and volume are maintained (Takahashi et al., 2000). When expressed in *Xenopus* oocytes, NKCC2 mutants show impaired sodium transport activities compared with wild type (WT) (Starremans et al., 2003). In contrast, abnormally enhanced NKCC2-mediated NaCl reabsorption is associated with hypertension in humans (Aviv et al., 2004; Jung et al., 2011; Gonzalez-Vicente et al., 2019) and animal models (Haque et al., 2011; Ares et al., 2012; Capasso et al., 2005; Sonalker et al., 2007) of spontaneous and salt-sensitive hypertension (Ortiz et al., 2003). Increased NKCC2 activity is thought to impair pressure-natriuresis resetting blood pressure to a higher level to maintain appropriate NaCl excretion. However, there have been very few studies that directly test this hypothesis. Lately, there has been great interest in identifying factors that regulate NKCC2 activity and provide a physical scaffold for NKCC2 trafficking, signaling, and apical membrane residence-time, since any of these pathways could be involved in salt-sensitive hypertension. A comprehensive molecular model for NKCC2 interactions that control these pathways is just starting to emerge but requires additional research and development of new methods to advance this faster.

The activity and expression levels of NKCC2 in TAL are regulated by multiple hormones including vasopressin (AVP), parathyroid hormone (PTH), calcitonin, glucagon, and catecholamines acting as agonists. In contrast, prostaglandins and extracellular Ca^2+^ act on the inhibitory component to counterbalance the physiological regulation of NKCC2 (Gunaratne et al., 2010). Transgenic rats with TAL-specific suppression of vasopressin V2 receptors (V2R) showed markedly impaired urinary concentration (Mutig et al., 2016). These data suggest AVP acts through the V2R to maintain the NKCC2 activity (Mutig et al., 2016). In addition, other factors besides expression levels, including alternative splicing, NKCC2 trafficking at the apical surface, protein-protein interaction, protein turnover and phosphorylation/dephosphorylation determine overall NKCC2-mediated NaCl reabsorption in TALs (Ares et al., 2011). Alternative splicing of the variable exon 4 of the *SLC12A1* gene gives rise to the full-length isoforms of the NKCC2: NKCC2A, NKCC2B, and NKCC2F (Castrop and Schnermann, 2008). These splice isoforms differ from the localization and their characteristics, including ion affinities, transport kinetic, and distribution patterns along with the TAL (Castrop and Schnermann, 2008; Carota et al., 2010). Localization of NKCC2 splice isoforms and their characterization are discussed elsewhere (Castrop and Schießl, 2014; Schießl and Castrop, 2015). Like other members of the CCCs, post-translational modification is essential to regulate NKCC2 activity via phosphorylation/dephosphorylation at the N-terminus of threonine and serine residue (Gimenez, 2006; Richardson et al., 2011). Kinases such as SPS1-related proline/alanine-rich kinase (SPAK, *STK39*) and oxidative stress-responsive kinase 1 (OSR1, *OXSR1*) has been reported directly phosphorylate the regulatory sites Thr95, Thr100, and Thr105 whereas Protein Kinase A phosphorylates Ser126 in the N-terminus tail of NKCC2, significantly increasing NKCC2 activity (Richardson et al., 2011). Phosphorylation at any of these sites could increase NKCC2 activity in Dahl salt sensitive animals where abnormally elevated salt reabsorption in the TAL was reported on normal or high salt diets. Increased NKCC2 in the apical surface and phosphorylation at Thre96,101 activity and chloride reabsorption have been found in these hypertensive rats, indicating that NKCC2 overactivation may be involved in hypertension (Alvarez-Guerra and Garay, 2002). However, the molecular mechanisms and genes leading to enhanced NKCC2 activity are not clear.

NKCC2 is the primary pharmacological target of loop diuretic drugs used worldwide to treat edematous states (Caceres and Ortiz, 2019). These loop diuretics including bumetanide, furosemide and torsemide are all powerful blockers of ion transport through NKCC2 (Zhao et al., 2022), but they are not specific an also potently inhibit NKCC1 (SLC12A2). In the past 2 decades, substantial effort has been made to understand the molecular mechanisms regulating the co-transporter since they could be critical to controlling blood pressure under physiological or pathological conditions (Caceres and Ortiz, 2019). Several recent reviews can provide an overview of NKCC2 regulation (Ares et al., 2011; Caceres and Ortiz, 2019; Mutig, 2017). Even though NKCC2 is of great importance in renal function, the molecular mechanisms by which NKCC2 expression is regulated in health and disease still needs to be determined. In this review, we will mainly discuss recent developments on molecular mechanisms of NKCC2 regulation, including apical trafficking of NKCC2 to the apical membrane and phosphorylation, protein-protein interactions with regulatory factors, and ubiquitination. These new pathways open exciting new avenues to modulate NKCC2 activity which would eventually evolve to controlling blood pressure (BP).

### Protein trafficking of NKCC2 to the apical membrane

Our laboratory previously showed that a small portion (3%–5%) of NKCC2 is expressed at the surface of the apical membrane (Ortiz, 2006), the rest being in an intracellular fragment (Nielsen et al., 1998). It is tightly regulated *via* trafficking into and out of the membrane, which is maintained by exocytosis, endocytosis, and recycling (Ares et al., 2012; Ortiz, 2006; Ares et al., 2008; Ares and Ortiz, 2010; Caceres et al., 2009; Caceres et al., 2014a; Caceres et al., 2016; Jaykumar et al., 2016). Little was known about the regulation of NKCC2 by the proteasome or lysosomes after internalization. However, recent work has suggested an additional mechanism that acts on the degradation of the internalized NKCC2 pool to regulate NKCC2 at the apical surface (Ares, 2019). Furthermore, the degradation of internalized NKCC2 is enhanced by the second messenger, cyclic guanosine monophosphate (cGMP), *via* the ubiquitin-proteasome system (Ares, 2019). A recent study from our group indicated that cullin-RING E3 ubiquitin ligase (CRL) complex may be involved in the cGMP-dependent increase in NKCC2 ubiquitination in TALs (Ares, 2023). Ares et al. argued that the neuronal precursor developmentally downregulated protein 8 (Nedd8) is responsible for CRL complex activity. Moreover, Cullin-associated and neddylation-dissociated 1 protein (CAND1) protein is also required for CRL activity. Inhibition of CAND1 by using a pharmacological inhibitor enhances baseline NKCC2 ubiquitination and exacerbated the cGMP-dependent increase in NKCC2 ubiquitination. These data suggested that cGMP-dependent ubiquitination of NKCC2 is regulated by a CRL complex (Ares, 2023). However, the detailed process of degradation and how ubiquitin dependent regulation of NKCC2 levels affect NKCC2 trafficking has yet to be studied. These observations are fascinating because they suggest that apical trafficking of NKCC2 and protein turnover are maintained by inhibitory signaling (cGMP, NO) that puts a brake on stimulatory signaling like cAMP and reactive oxygen species (Saez et al., 2018). These signaling cascades are fundamental for the regulation of TAL-mediated NaCl absorption by hormones, neurotransmitters, and variation in luminal flow. In addition to hormonal-mediated signaling, NKCC2 may be regulated by physical factors such shear stress and flow. Recent evidence shows that luminal flow increases superoxide generation by NADPH oxidases, which then stimulates NKCC2-mediated Na absorption by the TAL (Haque and Ortiz, 2019). Luminal flow-stimulated NKCC2 activity was blocked by tetanus toxin, a protease that blocks vesicle fusion and trafficking by cleaving vesicle-associated membrane fusion protein VAMP2 and 3 (Caceres et al., 2016; Caceres et al., 2014b). These data suggest that multiple pathways are involved in the control of NKCC2 trafficking. How the signaling form these pathways are integrated *in vivo*, in conditions of varying flow, luminal NaCl and hormonal state, is still poorly understood. Supposedly, there are trafficking pathways that more “constitutive” or less sublet to regulation (Caceres et al., 2016), whereas there are trafficking pathways that strictly dependent on specific signals. For example, ubiquitin-dependent modulation of NKCC2 levels may specifically affect post-endocytic or recycling pathways, while signaling that requires fast stimulation act on exocytosis from a “reserve” pool of vesicles or from am apical recycling compartment.

Recent reports suggested that melanoma-associated antigen D2 (MAGED2) is a novel regulator of NKCC2, a gene identified by whole-exome sequencing in families affected by transient antennal Bartter’s syndrome with polyhydramnios (Laghmani et al., 2016). Interestingly, patients with a mutation in MAGED2 have reduced surface expression of NKCC2 with more prominent NKCC2 in the cytoplasm and colocalized with an endoplasmic reticulum marker (Laghmani et al., 2016). Using coimmunoprecipitation of MAGED2, the authors demonstrated that MAGED2 interacts with Hsp40 and G-protein Gs-alpha, which opens new possibilities for control of NKCC2 activity (Laghmani et al., 2016). This lab also showed that Hsp40 might protect NKCC2 from endoplasmic reticulum-associated degradation (ERAD) and regulate NKCC2 exit from the endoplasmic reticulum in OK and HEK-293 cell lines (Zaarour et al., 2009; Seaayfan et al., 2016; Seaayfan et al., 2022). Gs-alpha activates cAMP production by adenylyl cyclases which we showed stimulate NKCC2 exocytic delivery and recycling in TALs (Caceres and Ortiz, 2019). cAMP, is also known to increase the total expression of NKCC2, therefore it is plausible that one of the main mechanism to increase NKCC2 expression is to prevent its ERAD and shuttle a greater fraction of NKCC2 to the TGN for continued maturation. This connection between NKCC2, cAMP, MAGED2 and ERAD is a very interesting observation that needs to be studied further, in particular, to understand molecular mechanisms that control NKCC2 expression at the apical surface of TAL.

Moesin, a protein belonging to the ezrin family, interacts with c-NKCC2 (NKCC1 backbone with swapped NKCC2 C-terminus driving apical trafficking) in LLC-PK1 cells. Silencing of moesin by short interfering RNA significantly reduced the expression of c-NKCC2 at the apical surface and accumulated it in the intracellular fraction (Carmosino et al., 2012a). In contrast, another group found the opposite observation in moesin knock-out mice. The authors demonstrated that knock-out mice have increased surface expression of NKCC2 at the apical surface of TAL than control (Kawaguchi et al., 2018). Moreover, the deletion of moesin seem to impair NKCC2 endocytosis, based on subcellular fractionation studies, and decrease the association with lipid rafts. These data suggested that moesin regulates the internalization of NKCC2 (Kawaguchi et al., 2018). However, the precise mechanism remains unclear. Moesin binds over 300 proteins (BioGrid database), many of which are involved in trafficking and actin crosslinking. Therefore, it is likely that deletion of moesin from whole animal has multiple pleiotropic effects and experiments should be designed to specifically increase or decrease expression of moesin in TALs to study NKCC2 trafficking.

In addition to the dynamic balance between NKCC2 apical trafficking and post-translational modifications, there are several dietary factors, metabolites, and hormones that affect NKCC2 activity by enhancing/decreasing the apical trafficking of NKCC2 in the TAL. To understand new dietary factors that affect NKCC2, our laboratory studied the effects of a high salt diet and the impact of dietary sugars, particularly fructose, because of its increased consumption of the western diet. In addition, elevated fructose consumption together with a high salt diet indices hypertension in rodents and has been linked to the development of hypertension in patients (Cabral et al., 2014; Huang et al., 2017). We specifically found that fructose, but not glucose, directly added to suspensions of medullary TALs, increased surface NKCC2 expression. Fructose added to the lumen or bath of isolated perfused TALs also increased NKCC2 activity (Ares et al., 2019). However, the specific signaling activated by fructose, and how it affects NKCC2 trafficking to increase NKCC2 activity is unclear and requires additional research.

### Protein-protein interactions regulate NKCC2

A handful of proteins were identified as NKCC2-binding partners using mass spectrometry and liquid chromatography in the last decades (Ares et al., 2011; Caceres and Ortiz, 2019; Mutig, 2017). The C-terminus of NKCC2 is unique and contains the conserved domain of dileucine-like motifs, which is not present in NKCC1, NCC, or other mammalian proteins (Gamba and Friedman, 2009) and confers NKCC2 apical targeting, such that when deleted, NKCC2 traffics to the basolateral membrane (Carmosino et al., 2008; Carmosino et al., 2012b; Carmosino et al., 2010). This dileucine-like motif is responsible for apical trafficking and its recognition by other interacting proteins of NKCC2 (Zaarour et al., 2009; Carmosino et al., 2008; Zaarour et al., 2012). The ability of regulatory proteins to bind a specific region of the carboxyl-terminus of the NKCC2 domain of approximately 70 amino acids. These interacting proteins regulate NKKC2 by either increasing the expression level of NKCC2 at the apical surface, such as MAL (Carmosino et al., 2010), VAMP2 (Caceres et al., 2014a), VAMP3 (Caceres et al., 2016), Annexin A2 (Dathe et al., 2014), moesin (Carmosino et al., 2012b) or decreasing the surface expression of NKCC2, such as Aldolase B (Benziane et al., 2007), SCAMP2 (Zaarour et al., 2011), OS9 (Seaayfan et al., 2016), ALMS1 (Jaykumar et al., 2018) and ACTN4 (Maskey et al., 2019).

Our laboratory identified vesicle fusion proteins VAMP2 and VAMP3 as important interacting proteins of NKCC2 involved in physiological NaCl reabsorption (Caceres et al., 2016). VAMP2 mediates cAMP-stimulated NKCC2 exocytic delivery and apical surface expression in TALs (Caceres et al., 2014a). A recent report suggested that moesin interacts with NKCC2 and is also involved in exocytic delivery of NKCC2. This was demonstrated by knocking down moesin with short interfering RNA, which significantly reduced the expression of NKCC2 at the apical surface and accumulated it in the intracellular fraction (Carmosino et al., 2012a). It is not known if moesin interacts with the SNARE protein pathway in the kidney. The BioGrid database does not show any interaction of moesin with SNAREs, suggesting that it may affect NKCC2 trafficking primarily by modulating signaling.

Recently, our laboratory identified Alström Syndrome 1 (ALMS1) as a novel protein that interacts with a specific region of the carboxyl terminus of NKCC2 (Jaykumar et al., 2018) at a sequence its binding partner recognizes. We investigated the role of ALMS1 in NKCC2 regulation in the apical surface of TAL and blood pressure control in rats with genetic deletion of ALMS1 and after *in vivo* shRNA-mediated gene silencing in TAL *in vivo*. We observed that the expression of NKCC2 was higher in the apical surface of TALs in ALMS1 KO rats, leading to increased NKCC2 activity. Importantly, the rate of NKCC2 endocytosis was slower in ALMS1-deleted rats, and ALMS1 co-localized with internalized NKCC2-containing vesicles (Jaykumar et al., 2018), indicating that ALMS1 is involved in some part of the endocytic pathway. It remains to be determined, precisely at which step of endocytosis ALMS1 interacts with NKCC2. We found that ALMS1 bound proteins from both the clathrin- and lipid raft-dependent pathways in TALs (Jaykumar et al., 2018). We also showed that NKCC2 undergoes endocytosis by both clathrin-, and lipid raft-mediated mechanisms (Ares and Ortiz, 2012) suggesting that ALMS1 may act at early endocytic steps of vesicle formation to mediate NKCC2 endocytosis. However, these specific questions need further study and the molecular mechanisms by which ALMS1 mediates endocytosis is unclear. In that paper, we also found that ALMS1 bound ACTN4. ACTN4 mutations cause focal glomerulosclerosis (FSGS) protein, but its function in the nephron was unknown. Our unpublished data show that *in vivo* silencing of Actinin-4 (ACTN4) in TALs caused NKCC2 accumulation at the apical surface of TALs, suggesting it also mediates NKCC2 endocytosis in TALs (Maskey et al., 2019). In addition, specific *in vivo* silencing of ACTN4 in TALs using CRISPR/Cas9, increased surface NKCC2 expression and enhanced NaCl reabsorption by TALs (maskey and Ortiz, 2022; Maskey et al., 2024). This is important because SNPs in ACTN4 are associated to hypertension in the general population (Chiang et al., 2014) and it is possible that ACTN4 is also involved in hypertension by modulating the ALMS1-NKCC2 interaction or acting independently on ALMS1 on NKCC2 to mediate its endocytosis. These mechanisms have not been directly studied but require clarification given that both ALMS1 and ACTN4 may be associated to hypertension in patients through the regulation of NKCC2.

### NKCC2 is regulated by phosphorylation

To date, 20 residues in the amino and carboxy terminus in human NKCC2 have been reported to be phosphorylated in high throughput and low throughput published reports, according to Phosphosite.org. Details about the characterization of these sites and physiological relevance are described in previous reviews (Ares et al., 2011; Schießl and Castrop, 2015; Caceres and Ortiz, 2019; Mutig, 2017) and Phosphosite.org. It is important to stress the significant physiological relevance of phosphorylation at T100 and T105 residues (equivalent to T96 and T101 in mice and rats) in the amino terminus of NKCC2, which have been shown to be essential for baseline transport activity (Giménez and Forbush, 2005; Giménez and Forbush, 2007; Hannemann and Flatman, 2011). Mutation of either T95 or T100 reduced NKCC2 phosphorylation, and mutation of both threonines, abolished phosphorylation detected with a T95/T100 antibody (Richardson et al., 2011). Importantly, phosphorylation at these residues is detectable, at baseline unstimulated conditions, in thick ascending limbs or kidney lysates of mice and rats (Giménez and Forbush, 2005; Giménez and Forbush, 2003).

Another well described phosphorylated site in NKCC2 is S126 at the amino terminus. Fraser et al. (2007) described AMP-activated protein kinase (AMPK) phosphorylate NKCC2 on S126 *in vitro*. They also demonstrated that mutating the S126 site significantly reduced rubidium influx under isotonic conditions in *X. laevis*. This data suggests that p-S126 maintains NKCC2-mediated transport under basal conditions in oocytes. S126 is also a target of another kinase, protein kinase A (PKA), as reported by Gunaratne et al. (2010), with the annotation that AMPK has a much lower ability to phosphorylate S126 since it has a preference for methionine or leucine. They also reported S874 as a target for PKA *in vivo*, and virtually nothing is known about the function of this site. STE20/SPS1-related proline-alanine-rich protein kinase (SPAK) and oxidative stress-responsive kinase-1 (OSR1) were the kinases found to phosphorylate NKCC2 at the T96 and T101 (Piechotta et al., 2002). They are the most recognized and studied targets of WNK kinases. WNK1 and WNK4 interact through their RFx [V/I] motif with the highly conserved CCT domain of SPAK and OSR1 (Vitari Ac Fau - Deak et al., 2005; Moriguchi et al., 2005). Then, WNK1 and WNK4 phosphorylate T-loop threonine within SPAK (T233) and OSR1 (T185) kinase domains to activate them (Piechotta et al., 2002; Vitari Ac Fau - Deak et al., 2005; Moriguchi et al., 2005). In turn, SPAK/OSR1 phosphorylate CCCs to activate them (NCC, NKCC1, NKCC2) or deactivate them (KCC3) (Piechotta et al., 2002; Gagnon and Delpire, 2010; Chiga et al., 2008).

It has been shown that activated SPAK and OSR1 phosphorylate NKCC2 at a stoichiometry of ∼0.3 and ∼0.7 mol of phosphate per mol of NKCC2 *in vitro*, respectively (Richardson et al., 2011). SPAK and OSR1 belong to the Sterile20-related protein kinase family and the GCKs subfamily, which has a 5′ or amino-terminal catalytic domain (Richardson et al., 2011; Boyce and Andrianopoulos, 2011; Gagnon and Delpire, 2012). The National Center for Biotechnology Information lists the gene STK39 encoding human SPAK and OXSR1 encoding for human OSR1, which share 67% homology in their amino acid sequence. The Human Protein Atlas reports medium to high protein expression of SPAK in the brain, respiratory system, gastrointestinal tract, liver, gallbladder, liver, kidney, and male and female tissue. A wider distribution is detected for OSR1. In the kidney, SPAK is localized in the medullary and cortical TAL, the DCT (Rafiqi et al., 2010) and lowere levels in collecting ducts, while OSR1 is expressed along the nephron (Yang et al., 2010; Mercier-Zuber and O'Shaughnessy, 2011). STK39 has been identified as a hypertension susceptibility gene in Amish, Han Chinese Europeans, and East Asian subjects (Wang et al., 2009; Chen et al., 2012; Xi et al., 2013). but no such association has been found for OSR1. Because its high homology, similar tissue distribution and the fact that OSR1 can also be activated by WNKs and can phosphorylate CCCs, OSR1 is studied in parallel with SPAK. It is worth mentioning that an indication of activated SPAK/OSR1 is the phosphorylation of S383 and S325, respectively (Richardson and Alessi, 2008; Susa et al., 2012). Given the high homology between the kinases, all antibodies directed to the phosphorylated forms recognize both SPAK and OSR1, most studies in the kidney measure both SPAK and OSR1 phosphorylation at this site as an index of activation. The differential effects of SPAK and OSR1 in different nephron segments still require additional study.

Over the past 20 years since Piechotta’s publication, whose valuable contribution was providing a link between monogenic BP syndromes and the CCCs, several knock-in and knock-out animal models have been generated to explore the regulation of the CCCs phosphorylation by SPAK and OSR kinases *in vivo* (Piechotta et al., 2002). Table 1 summarizes the experimental models and findings in them. Different genomic manipulation was made to disrupt either SPAK or OSR1 genes. Notably, there are more models for SPAK than OSR1, the former are real animal knock-out or knock-ins.

**TABLE 1:** SPAK and OSR1 genetic edition effect on NKCC2 phosphorylation.

Experimental model	Generation of experimental model	Phenotype*	NKCC2 and NCC phosphorylation*	SPAK/OSR1 phosphorylation	References
Knock-in mouse SPAK^243A/243A^	Mutation Thr243 in SPAK protein	Hypotensive on normal diet Mild hypomagnesemia and moderate hypocalciuria on normal diet (0.3% Na+) Mild hypokalemia, hyperaldosternonism, ↑plasma corticosterone on low Na diet (0.03% Na+)	Total NKCC2 — ↓37% pNKCC2(T96) — ↓82% Total NCC — ↓30% pNCC(T53) — ↓61% pNCC(T58) — ↓86% pNCC(S89) — ↓78%	NS	Rafiqi et al. (2010)
SPAK−/− mice	Deletion of exons 9 and 10 of Stk39	hypotensive, hyperaldosteronism, hypocalciuria, mild hypokalemia (Gitelman Syndrome) normal diet	Total NKCC2 — ↑130% pNKCC2(T96) — ↑360% Total NCC — ↓58% pNCC(T58) — ↓76% pNCC(T71) — ↓63%	Total OSR1 — unchanged pOSR1— ↑93%	Yang et al. (2010)
Global OSR1^+/−^mice KSP-OSR1−/− mice	NS	Global OSR1^+/−^ hypotensive on normal and low Na diet (0.05% Na+)	Total NKCC2 — unchanged pNKCC2(T96) — ↓28% Total NCC — ↑35% pNCC(T58) — ↑ 21% pNCC(T71) — ↑56% (Normal diet 0.4% Na+)	Total OSR1 — ↓25% pOSR1— ↓31% KSP-OSR1−/− Total SPAK — unchanged pSPAK —↑28% (Normal diet 0.4% Na+)	Lins et al. (2021)
		KSP-OSR1−/− normotensive on a normal diet (0.4% Na+) and hypotensive low Na diet (0.05% Na+), hypokalemia, ↑FEK, hypercalciuria, ↓urine osmolarity (Bartter Syndrome like)	Total NKCC2 — unchanged pNKCC2(T96) — ↓68% Total NCC — ↑58% pNCC(T58) — ↑38% pNCC(T71) — ↑27% (Normal diet 0.4% Na+)	Total SPAK — ↑30% pSPAK —↑38% (Normal diet 0.4% Na+)	
SPAK−/− mice	Duplication of exon 6	Normotensive on a normal diet (0.49%), hypotensive on a low Na diet (0.01%NaCl) Trend to hypokalemia and hypomagnesemia, hypocalciuria on normal diet, ↑PRA	Total NKCC2 — unchanged pNKCC2(T96,101) — ↑400% Total NCC — ↓90% pNCC(T53) — ↓90% (Normal diet 0.4% Na+)	Total OSR1 — unchanged pOSR1— **↑**216% (Normal diet 0.4% Na+)	McCormick et al. (2011)
SPAK−/− mice	Duplication of exon 6	Hypotensive, ↑FEK, ↑FEMg, ↑BUN, aldosterone Normal Na diet ↑U_Na_V on a low Na diet	Total NKCC2 — unchanged pNKCC2(T96,101) — ↑225% Total NCC — ↓78% pNCC(T58) — ↓79%	Total OSR1 — unchanged pOSR1 — NS	Grimm et al. (2012)
SPAK−/−•ksOSR1−/− (Double knock-out)	Pax8-rtTA/LC1 system Exons 1–4 of OXSR1	**Hypotensive, hypokalemia ↑BUN and hematocrit on a low Na diet	Total NKCC2 — unchanged* pNKCC2(T96,101) — ↑400%*, 1,200%** pSer126 — ↓50%** Total NCC — unchanged** pNCC (T53) — ↓80%**	—	Ferdaus et al. (2016)

*Respect to WT, littermates, ** Respect to SPAK−/− mice. NS, not studied/not reported; BUN, blood urea nitrogen; FEK, fractional excretion of potassium; FEMg, fractional excretion of magnesium; PRA, plasma renin activity; U_Na_V, urinary sodium excretion.

Genetic deletion of SPAK alone in mice results in a phenotype characterized by hypotension, hypocalciuria and hypokalemia, known as Gitelman-like syndrome on a regular sodium diet (Rafiqi et al., 2010; Yang et al., 2010; Grimm et al., 2012), or low sodium diet (McCormick et al., 2011). In all models, NCC phosphorylation at T53, T58, T71, and/or S81 are reduced, varying between 61% and 90% concerning WT and a reduction of total NCC. For NKCC2, the results are disparate. Rafiqi’s group knock-in mouse is the only one reporting a decrease in total and phospho-NKCC2 (Chiga et al., 2008), whereas other groups report no change, which may be explained under Dr. McCormick’s hypothesis of various SPAK isoforms. Another interesting fact to note is that total OSR1 expression is unchanged during SPAK deletion while OSR1 phosphorylation rises between 93% and 216%. These data suggest that SPAK plays a fundamental role in the DCT and a less prominent role in TAL when whole kidney homogenates from mice are used for blotting. It is also possible that SPAK deletion drives compensatory phosphorylation of OSR1 that is different in TALs or DCT. Opposed to these findings, our group generated a SPAK KO rat model on a Dahl Salt sensitive background that exhibits lower blood pressure than the WT and a 60% reduction of T96,101 NKCC2 phosphorylation in suspensions of medullary TALs (unpublished data). One possible explanation is the antibody specificity used in mouse studies, which may recognize both pNCC and pNKCC2 in whole kidney lysates. Another explanation could be that different regulatory pathways operate SPAK and OSR1 in the rat *versus* mice, requiring further study. Concerning the single genetic deletion of OSR1, only one model was reported. This may arise from the difficulty of disrupting OSR1, since OXSR1 absence is lethal in embryos and only heterozygous or kidney-specific animals are viable (Rafiqi et al., 2010; Yang et al., 2010; Lins et al., 2021) bred global heterozygous OSR1 (+/−) and kidney-specific knockouts (KSP-OSR1^−/−^). OSR1 (+/−) mice only showed relative hypotension with no electrolyte abnormalities, while the KSP-OSR1^−/−^ displayed Bartter-like syndrome and hypotension on a low sodium diet. Phosphorylation of NKCC2 was dramatically reduced (68%), but phospho-T58,71 NCC was increased, together with SPAK phosphorylation. A similar conclusion is drawn for OSR1 disruption, apparently being important for TAL NKCC2 phosphorylation in the absence of SPAK, accompanied by the compensatory phosphorylation of SPAK in the DCT.

McCormick et al. introduced the concept of different isoforms of SPAK with other effects in the distal nephron to explain the disparities in NKCC2 and NCC phosphorylation among the models (McCormick et al., 2011). The full-length SPAK is more abundant in the DCT and phosphorylates NCC, while the truncated form (absent kinase domain, ks-SPAK) is mainly expressed in the TAL and inhibits phosphorylation of NKCC2 (Park et al., 2013). So, when completely deleting SPAK, ks-SPAK is deleted, and NKCC2 phosphorylation increases. Grimm et al. further confirmed this hypothesis, adding that the hyperphosphorylation of NKCC2 may be further elevated due to AMPK since they found pAMPK was significantly increased in the medulla of SPAK^−/−^ mice (Grimm et al., 2012).

In most papers, authors measure the phosphorylation of T96 and T101 to indicate activity. However, Hannemann, Flatman et al. point out that an increase in phosphorylation does not always accurately reflect an increase in transport activity (and *vice versa*), as they found with calyculin. HEK-293 cells, stably expressing ferret NKCC1(fNKCC1) and ferret NKCC2 (fNKCC2), were treated with calyculin, causing a 5-fold increase in phosphorylation of fNKCC1 and fNKCC2, but only a 30% increase of Rb + transport in fNKCC1 and a small drop of Rb + transport in fNKCC2. Since an increase in phosphorylation may be due to phosphatase activity (e.g., calyculin A), experiments examining actual ion transport (Wang et al., 2009) Rb + uptake, Tl + influx, intracellular Cl, or Na with fluorescent dyes (such as SBFI) must be performed together with phospho-NKCC2 blotting.

Some other factors should be considered for an accurate and reproducible NKCC2 phosphorylation measurement in mice, especially those with a C57BL/6 mouse background. (Moser et al., 2021) tested different tissue processing protocols and found that the pNKCC2 band at 170 KDa became more intense when fresh kidney lysates were used compared to frozen samples. Also, using a lysis buffer impacted the intensity of band detection, with the DFLB buffer better than RIPA in obtaining a good signal intensity. However, one of the major problems they encountered was that the existing anti-p-NKCC2 antibodies were directed to the T96 and T101 in mouse models, but the C57BL/6J mouse NKCC2 lacks five amino acids 97–101 which may affect reactivity of some antibodies in mice. The second problem was the cross-reaction of existing anti-p-NKCC2 antibodies (pT212/T217 NKCC1 R5 a-Human, pT96/T101 NKCC2 9934 AP a-Rat, pT96/T101 a-Mouse) with the phosphorylated forms of NCC (T53 and T58) given the similarities in the phospho region. In response to this gap, they produced a new antibody directed to the amino acids surrounding the T96, YYLQ(p)TMDA. They thoroughly validated the new anti-p-NKCC2 antibody by comparing its specificity with previously reported anti-pNKCC2 and anti-p-NCC antibodies (Moser et al., 2021). This work stresses that simple technicalities, the use of whole kidney vs. dissected tubule suspensions or different strains may greatly impact the results.

SPAK and OSR1 are known targets of the With No Lysine Kinase 4 (WNK4) kinase pathway in the distal convoluted tubule. However, the effect of WNK4 in the TAL is still obscure, as many groups have reported different results, partly due to the cross-reaction of the pNKCC2 antibody with pNCC. Meoka et al. attempted to clarify the role of the WNK4-SPAK/OSR1 pathway on NKCC2 phosphorylation (Maeoka et al., 2024), with the new pT96-NKCC2 antibody. Wnk4^−/−^ deletion in mice did not lower pT96-NKCC2 abundance and minimally reduced the phosphorylated activated forms of SPAK and OSR1 (pSPAK/pOSR1) in the TAL. This last finding led the authors to ask why the kinases were still phosphorylated with Wnk4 deletion. To answer their question, they used immunofluorescence to detect Wnk1, which is highly expressed in the TAL and DCT, they found that Wnk1 was localized to apical side of the TAL, suggesting this isoform would be responsible for the SPAK/OSR1 phosphorylation, and not Wnk4.

### Hormones and other molecules modifying NKCC2 phosphorylation

Even though phosphorylation of T96,101 occurs at baseline conditions, it is enhanced by physiological stimuli. Many hormones that stimulate NKCC2 activity and phosphorylation, e.g., Arginine Vasopressin (AVP) (Rieg et al., 2013) and β-adrenergic receptor stimulation (Garg et al., 1992; Plato and Garvin, 2001; Haque et al., 2012), increase intracellular levels of the second messenger cAMP.

AVP is essential in body electrolyte homeostasis by controlling, in part, the kidney’s ability to reabsorb water in the distal nephron. However, in the TALs receptors to AVP, V2R are expressed (Nonoguchi et al., 1995), suggesting AVP has some stimulatory effect on NKCC2. Having this hypothesis in mind, Gimenez and Forbush (2003) tested an acute stimulation (1 h) with dDAVP in CD-1 mice; using the R5 antibody (an anti-phospho-NKCC1 antibody) they found dDAVP enhanced NKCC2 phosphorylation at T96,101 and also increased apical fraction in TALs. These data showed that AVP regulates NKCC2 activation but leaves doubt about the kinase-mediated phosphorylation. To answer this last question, Sarita et al. (2013), using a short dDAVP stimulation again, found that DAVP modulates the interaction of NKCC2 with SPAK isoforms. dDAVP reduces the binding of KS-SPAK and increases the binding of FL-SPAK. In addition, dDAVP-V2R stimulation with desmopressin mainly induces phosphorylation of SPAK but to a lesser extent of OSR1 in mTAL, leading to NKCC2 phosphorylation at T96/T101 (Saritas et al., 2013). To put another piece in the puzzle, Vallon’s group investigated the downstream pathway of AVP. They generated a knock-out for Adenylyl Cyclase 6 (AC6^−/−^) and tested the effect of dDAVP. The isoform AC6 accounts for part of the dDAVP-induced NKCC2 expression and phosphorylation at S126. AC6^−/−^ mice have a 50% reduction in total NKCC2 expression compared to wild-type animals. Treatment with dDAVP, increases NKCC2 abundance two-fold compared to wild-type mice (Rieg et al., 2013). AC6^−/−^ mice also have lower S126-NKCC2 phosphorylation in response to dDAVP after overnight water loading. AVP has also been involved in calcineurin-sorting protein-related receptor with A-type repeats (SORLA) activation of NKCC2 (Borschewski et al., 2016).

Another key axis regulating electrolyte and water homeostasis is the renin-angiotensin system (RAS). In the distal nephron, chronic Angiotensin II (Ang II) induces phosphorylation of NCC via the WNK4-SPAK pathway (van der Lubbe et al., 2012; Castañeda-Bueno et al., 2012). Ang II enhances Na reabsorption directly by increasing Na transport along the nephron, including the TAL (Silva and Garvin, 2008; Wu and Johns, 2002). 14 days infusion of Ang II seems to regulate NKCC2 expression in the cortex and medulla differentially; in the cortex, it increases total and p-T96,101NKCC2 but decreased total and doesn’t change p-T96,101 NKCC2 in the medulla (Nguyen et al., 2015). More prolonged infusion of Ang II (42 days) also increases total cortical NKCC2 expression (Lins et al., 2021). SPAK is also differentially regulated, prolonged Ang II causes a greater total and phosphorylated forms in the cortex but a lower expression in the medulla (Nguyen et al., 2015). These data suggest that SPAK may be involved in the phosphorylation of NKCC2 by Ang II. It is important to note that in those studies, whole kidney homogenates where used, and is possible that p T96-101 antibodies cross-reacted with pT53 in NCC.

Growth hormone (GH) has sodium-retaining properties partly mediated by insulin-like growth factor 1(IGF-1) (Haffner et al., 1990). GH mainly acts by regulating ENaC transcription (Kamenicky et al., 2008) and activation (Kamenicky et al., 2011), it also appears to activate NKCC2 in the medullary TAL indirectly. Administration of recombinant human GH to Wistar-Hannover rats significantly increased phosphorylation of T96,101-NKCC2 in the inner stripe of outer medulla thick ascending limbs. Additionally, increased NKCC2 expression was observed in the cortical region. The authors leave elusive the role of NKCC2 activation on the anti-natriuretic effect of GH.

Uromodulin, or Tamm-Horsfall protein (THP), is highly expressed in the kidney with the highest mRNA levels in the cortical TAL followed by the medullary TAL where it localizes with NKCC2 at the apical side (Nielsen et al., 1998; Bachmann et al., 1985). It has been shown to play a minor role in water homeostasis and salt reabsorption. THP knockout animals showed lower levels of pNKCC2 compared to WT animals. Furthermore, a blunted sodium excretion was observed when these mice were treated with furosemide. Also, THP seems involved in NKCC2 response to chloride depletion, which suggests SPAK/OSR1 kinases mediate the interaction between THP and NKCC2 (Mutig et al., 2011). Following these findings, transiently transfected HEK 293 with uromodulin led to an increase of NKCC2 phosphorylation at T96,101 and its activity. In transgenic TgUmod wt/wt mice overexpressing THP, p-T243 SPAK and T185 OSR1 were increased, supporting the upregulation of these kinases (Trudu et al., 2013). However, whether the mechanism of NKCC2 regulation by uromodulin is direct or indirect is unclear. We could not immunoprecipitate UMOD with endogenous NKCC2, and we have never identified it as interacting with the amino-terminus or carboxyl terminus (unpublished observations), suggesting that UMOD may control cAMP or WNK signaling in TALs rather than binding NKCC2.

Mouse protein-25 (MO25) has been found to modulate SPAK and OSR1 activity, hence NKCC2 phosphorylation. MO25 isoforms, alfa, and beta, induce 100-fold activation of SPAK/OSR1, which enhances their ability to phosphorylate NKCC2 (and other targets such as NKCC1 and NCC) on the canonical residues, T95,101 but also the sites Thr118 and Ser120 (Filippi et al., 2011).

Calcineurin is a ubiquitous serine/threonine protein phosphatase that seems to regulate NKCC2 activity since cyclosporine, a calcineurin inhibitor, induces hypertension and impairs Na reabsorption in medullary TALs (Tumlin and Sands, 1993). Even though calcineurin has the potential to dephosphorylate NKCC2 at T96-101 directly, this has not been shown. Cyclosporin-treated rats (25 mg/kg) developed sodium retention, low urine osmolarity after 7 days, and increased NKCC2 expression (Esteva-Font et al., 2007). Cyclosporin increases phosphorylation of SPAK/OSR1 hence phosphorylation of NKCC2 at T96,101 (Borschewski et al., 2016). The Mutig group, previously showed that genetic deletion of SORLA is associated with reduced NKCC2 phosphorylation (Reiche et al., 2010). Later, they linked SORLA and calcineurin in the same signaling pathway in the TAL to modulate NKCC2 phosphorylation by modulating SPAK (Borschewski et al., 2016).

As we described above, fructose increases NaCl transport via NKCC2 in rat TALs by increasing the surface expression of NKCC2. Interestingly, fructose, added acutely (30 min) did not affect NKCC2 phosphorylation (Ares et al., 2019). Later, our laboratory further explored the effect of fructose and high salt diet on NKCC2. We found that chronic feeding of fructose (20% in drinking water) with or without high salt diet increased NKCC2 phosphorylation at T96,101 and S126, compared to baseline or salt-alone groups (Ares and Ortiz, 2015). This was supported by transcriptome profiling showing upregulation of SPAK and AMPK pathways genes (Kassem et al., 2017).

Accumulating evidence links an enhanced p-T96,101 to increased salt reabsorption in salt-sensitive hypertension. Micro-perfusion experiments in Dahl Salt-sensitive rats (Dahl SS) show enhanced absorption by the TAL exclusively (Kirchner, 1990; Hawk and Schafer, 1991; Kirchner et al., 1995; García et al., 1999; Ares et al., 2012) reported that NKCC2 phosphorylation at T96,101 is increased 5-fold in Dahl SS compared to salt-resistant rats on a regular salt diet (Roman and Kaldunski, 1991), and this was independent of the hypertension observed in these rats during high salt. Additionally, Dahl SS has a 60% increase of p-SPAK/OSR1, suggesting the enhanced NKCC phosphorylation is due to enhanced activity of SPAK and/or OSR1. Similarly, in Milan hypertensive (MHS) rats, phosphorylation levels of NKCC2 were significantly increased together with S325-SPAK increase phosphorylation (Carmosino et al., 2011). It remains to be determined whether enhanced NKCC2 phosphorylation, trafficking and activity are secondary to SPAK, OSR1 or other kinases and which are the intrinsic mechanisms that maintain an abnormally elevated NKCC2 even during high salt diet. In addition, there is little data on NKCC2 regulation in patients during hypertension, acute or chronic kidney disease and the contribution of this pathways in human disease should continue to be studied.

## Conclusion

**FIGURE 1:**
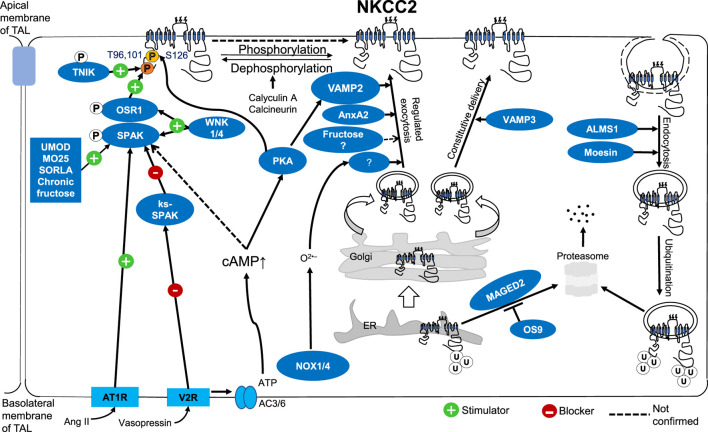
Molecular mechanism of NKCC2 regulation by trafficking, protein-protein interactions, and phosphorylation at the apical surface of TALs.

Altogether, recent data highlighted the critical steps of NKCC2 trafficking. Most likely, it controls the regulation of NKCC2 activity in the TAL. The molecular mechanism of NKCC2 trafficking at the apical membrane of the TAL has become a significant interest among researchers. We discussed how NKCC2 activity is controlled by trafficking, protein-protein interactions, and phosphorylation (Figure 1). Any gene or interacting protein that affects NKCC2 trafficking, or its phosphorylation could change the expression level of NKCC2 at the apical surface of TAL, or its activity which may affect salt reabsorption and BP control.
